# Impacts of Population Structure and Analytical Models in Genome-Wide Association Studies of Complex Traits in Forest Trees: A Case Study in *Eucalyptus globulus*

**DOI:** 10.1371/journal.pone.0081267

**Published:** 2013-11-25

**Authors:** Eduardo P. Cappa, Yousry A. El-Kassaby, Martín N. Garcia, Cintia Acuña, Nuno M. G. Borralho, Dario Grattapaglia, Susana N. Marcucci Poltri

**Affiliations:** 1 Instituto de Recursos Biológicos, Centro de Investigación en Recursos Naturales, Instituto Nacional de Tecnología Agropecuaria (INTA) and Consejo Nacional de Investigaciones Científicas y Técnicas (CONICET), Hurlingham, Buenos Aires, Argentina; 2 Department of Forest and Conservation Sciences, Faculty of Forestry, The University of British Columbia, Vancouver, British Columbia, Canada; 3 Instituto de Biotecnología, Centro de Investigación en Ciencias Veterinarias y Agronómicas, Instituto Nacional de Tecnología Agropecuaria (INTA), Hurlingham, Buenos Aires, Argentina; 4 Private Consultant, Cartaxo, Portugal and Centro de Estudos Florestais, Instituto Superior de Agronomia, Universidade Técnica de Lisboa, Lisboa, Portugal; 5 EMBRAPA Genetic Resources and Biotechnology and Genomic Sciences Program, Universidade Católica de Brasília, Brasilia DF, Brazil; National Taiwan University, Taiwan

## Abstract

The promise of association genetics to identify genes or genomic regions controlling complex traits has generated a flurry of interest. Such phenotype-genotype associations could be useful to accelerate tree breeding cycles, increase precision and selection intensity for late expressing, low heritability traits. However, the prospects of association genetics in highly heterozygous undomesticated forest trees can be severely impacted by the presence of cryptic population and pedigree structure. To investigate how to better account for this, we compared the GLM and five combinations of the *U*nified *M*ixed *M*odel (***UMM***) on data of a low-density genome-wide association study for growth and wood property traits carried out in a *Eucalyptus globulus* population (*n* = 303) with 7,680 Diversity Array Technology (DArT) markers. Model comparisons were based on the degree of deviation from the uniform distribution and estimates of the mean square differences between the observed and expected *p*-values of all significant marker-trait associations detected. Our analysis revealed the presence of population and family structure. There was not a single best model for all traits. Striking differences in detection power and accuracy were observed among the different models especially when population structure was not accounted for. The ***UMM*** method was the best and produced superior results when compared to GLM for all traits. Following stringent correction for false discoveries, 18 marker-trait associations were detected, 16 for tree diameter growth and two for lignin monomer composition (S∶G ratio), a key wood property trait. The two DArT markers associated with S∶G ratio on chromosome 10, physically map within 1 Mbp of the ferulate 5-hydroxylase (F5H) gene, providing a putative independent validation of this marker-trait association. This study details the merit of collectively integrate population structure and relatedness in association analyses in undomesticated, highly heterozygous forest trees, and provides additional insights into the nature of complex quantitative traits in *Eucalyptus*.

## Introduction

Understanding genotype-phenotype relationships and discovering genes or genomic segments with roles in the control of complex traits are challenges of major biological and economic importance. Deciphering this connection is considered to be geneticists' ultimate goal to determine the genetic underpinnings of biological processes while enhancing directional selection strategies in improvement programs. The combined advances in molecular markers development, functional genomics, and analytical methods, made it possible to gain insights into the architecture of complex traits by identifying underlying genes and genomic segments involved in their control (i.e., number, magnitude, and their possible interaction) [1]. The once elusive promise of effectiveness of molecular markers in population improvement is gradually becoming a reality for model and non-model species including forest trees. Neale and Savolainen [2] presented a compelling argument supporting the feasibility to dissect complex traits in forest trees as well as pointing to some of tree breeding perceived drawbacks, such as long generation span, substantial resources dependency, and the size required for proper testing across a vast geographic representation. Most economically important tree species are still largely undomesticated, having predominantly an outcrossing mating system and very large natural populations. This is expected to result in high nucleotide diversity and low linkage disequilibrium [3], [4], thus making them suitable candidates for association genetics or linkage disequilibrium mapping [2].

However, in such wild populations genetic structure, such as the presence of provenance or racial effects and/or intra-racial spatial relatedness between trees may affect association genetics results [5], [6]. Overlooking these structures in the data has been considered as one of the main causes of spurious associations (i.e., false positives) [7]. To overcome such drawbacks, several methods have been developed and successfully implemented [8], [9], [10]. Among the many solutions to account for genetic structure, the ‘*U*nified *M*ixed-*M*odel’ (***UMM***) [11] offered a simple method whereby both sub-populations (i.e., races or provenances) and cryptic kinship structures can be accounted for in association genetics analyses. In summary, population and kinship structures are quantified using a model-based Bayesian clustering algorithm (STRUCTURE; [12]) and one of the pair-wise kinship estimation procedures (e.g., [13], [14]) are then included in the ***UMM*** model as fixed or random effects, respectively [11]. Yu *et al*. [11] demonstrated the value of accounting for population and/or family structures through the incorporation of genomic control and marker-based kinship in mixed model association testing using two samples: a family sample of 14 human families and a sample of 277 diverse maize inbred lines. From that initial work, effects of population and family structure on associations has been investigated in several agricultural crops such as *Glycine max* [15], *Hordeum vulgare* [16], *Brassica rapa* [17], *Triticum aestivum* [18], [19] to name a few.

To date, a number of association genetics studies have been reported for forest trees using either structured or unstructured populations. Various analytical methods have been applied to detect associations, including classical general linear model (GLM) with and without family structure [20], [21], ***UMM*** [22], [23], [24], [25], [26], [27], [28], [29], [30], [31], and quantitative transmission test for linkage disequilibrium (QTDT) [32]. In the absence of population and/or family structures, the inclusion of the **Q** (population structure) and/or **K** (family structure) matrices in the ***UMM*** is expected to unnecessarily remove parts from the error term's variation and degrees of freedom to the population and/or family effects. This results in changes in the mean square error term which ultimately affects the statistical power of the association tests.

Due to the lack of accessible genome-wide genotyping systems and given the generally low extent of linkage disequilibrium in forest trees, all these association studies were carried out based on the targeted analysis of polymorphisms in candidate genes. Only more recently genome-wide genotyping approaches have been applied in *Pinus taeda* [28] and *Pinus contorta* [33] for association mapping, and in whole-genome prediction studies in *Pinus taeda* [34], and *Eucalyptus* [35]. While candidate gene association studies are severely biased by the *a priori* choice of genes that are allegedly involved in trait control, genome-wide studies are unbiased in this respect, and therefore tend to better converge to the true genetic architecture of the complex traits investigated.

Recently, a high throughput Diversity Array Technology (DArT) marker platform was developed for species of *Eucalyptus* [36]. Briefly, DArT is a genotyping technique based on genome complexity reduction, followed by hybridization to spotted probe microarrays that offer a rapid, cost-effective and efficient methodology for high-throughput genome-wide marker analysis [37]. The operational array with 7,680 selected polymorphic DArT markers is highly transferable across *Eucalyptus* species [38], [39], and has provided unprecedented level of resolution for linkage mapping [40], [41], [42], QTL analysis [43], [44] and Genomic Selection [35]. Interestingly, a detailed genomic characterization of these DArT markers aligned to the annotated *Eucalyptus grandis* reference genome, showed that they preferentially target the gene space with 77% of them positioned at <1 kbp from the nearest gene model. Moreover, they display a largely homogeneous distribution across the genome, thereby providing gene-targeted genotyping and good coverage for genome-wide applications in association genetics and Genomic Selection (GS) [42]. Given these very special attributes, the DArT array offers a useful platform for association genetic testing across the genome, although the number of markers assayed (7,680) is still less than optimal for a more powerful full fledged genome-wide association study.

As part of the Biotech MERCOSUR project [45] 303 individuals from different open-pollinated progeny trials of *Eucalyptus globulus* core and intergrade populations were genotyped with the 7,680 DArT marker array. In the present study, our main objectives were: (1) to test the efficacy of including the **Q** and/or **K** matrices in the association genetics analyses for complex traits for growth and wood properties and (2) to evaluate and compare the efficacy of different association mapping models to avoid declaring false marker-trait associations based on the degree of deviation between the observed and expected p-values of marker-trait associations. Additionally, despite of the relatively limited size association mapping population we had access to, our study also contributes to the understanding of the genetic architecture of economically important traits in *Eucalyptus globulus*.

## Materials and Methods

### Ethics statement

The *Eucalyptus globulus* Argentinian trial belongs to the Instituto Nacional de Tecnología Agropecuaria (INTA) and no specific permits were required to carry out the study. The three *Eucalyptus globulus* Uruguayan trials belong to Mundial Forestación. All the available data from these three trials were provided by the company director Rogerio de Aguiar de Moraes under the agreement of the Biotech MERCOSUR project. *Eucalyptus globulus* is an exotic tree species in Argentina and Uruguay and is not protected or endangered.

### Plant material, phenotypic traits, and genotyping

A sample of 303 *Eucalyptus globulus* (Labill.) individuals growing at four separate trial sites was used in this study (Table 1). One trial was located in Argentina: Balcarce, Buenos Aires province (37° 45′ S, 58° 17′ W) (134 trees), and the other three in Uruguay (33° 51′ S, 55° 34′ W) (in total 169 trees). The mapping population included trees from 161 families belonging to seven native races in Australia, and two bulk collections from local landraces originated in Portugal and Chile. All seeds were open-pollinated, except two control-pollinated full-sib seedlots from the Portuguese land race, in the Argentinian trial. The number of sampled trees per family varied between 1 and 9. Eighteen families from four Australian races planted in the Argentinian trial were also present in the two Uruguayan trials.

**Table 1:** Description of progeny trials in the different sites, numbers of individuals phenotyped and genotyped and phenotypic trait means (see text for traits' abbreviations).

								Phenotypic trait means					
Trial	Location	Year of establishment	Initial number of tree	Genetic material a	Association population b	Measurement ages (yr)^c^	WP and DArTs trees d	DBH	PILO	Klason lignin	Total lignin	S∶G ratio	Extractives
**BALC**	Argentina	1995	4200	14 (250)	13 (129)	4/15	134	11.7	12.6	22.4	27.7	2.02	3.68
**GLO**	Uruguay	2000	9930	12 (169)	8 (34)	6/8	92	13.9	18.2	21.2	27.4	2.21	3.69
**JEE**	Uruguay	2000	2934	3 (51)	1 (15)	6/8	38	14.1	18.0	21.9	27.9	2.13	3.05
**PSEUDO**	Uruguay	2000	6516	16 (109)	13 (22)	6/8	39	13.7	17.3	21.9	28.0	2.16	3.51

a Number of provenances (number of open-pollinated families) in the trial.

b Number of provenances (number of open-pollinated families) sampled in the association population.

c Ages at measurements of diameter at breast height and Pilodyn penetration and wood properties.

d Number of trees measured for wood properties (WP) and genotyped for the association study.

Several growth and wood properties traits were measured at the four sites (see Lopez *et al*. [46] for details). However, measurements for every trait were not taken in all sites. However, diameter at breast height (DBH), Pilodyn penetration (an indirect measure of wood specific gravity), Klason and total lignin, lignin monomer composition (Syringyl∶Guaiacyl S∶G ratio), and extractives in ethanol (Extractives) were measured in all sites and used in this study. Diameter at breast height (1.3 m from the ground) was measured in centimetres when trees were 4 (Argentina) and 6 (Uruguay) years old on all surviving trees. Pilodyn penetration (PILO) was measured in mm using a 6 J Forest Pilodyn with a 2 mm diameter pin, in an east to west direction and without bark (a small section of bark at 1.3 m above the ground was removed prior to the PILO readings being taken), when trees were 4 (Argentina) and 6 (Uruguay) years old on all surviving trees. Wood chemical components were estimated using Near-Infrared (NIR) spectroscopy. Briefly, for wood chemical traits, cambium to cambium wood cores were removed at breast height from each tree in May 2010 (Argentina; 15-year-old) and in October 2008 (Uruguay; 8-year-old) and air-dried (Argentina' samples) or oven-dried (Uruguay' samples). The wood samples were ground to pass through a 1-mm screen, and NIR spectra were obtained by diffuse reflectance using a Bruker Optics Co. Multi Purpose Analyzer (MPA). Partial least squares regression (PLSR) was used for the evaluation of the NIR spectra (NIR-PLSR models) and for the calculation of the prediction models. Validation of these predictions was undertaken using conventional chemical assays from 15–22 independent samples from those used to develop the model. All models obtained were adequate for screening trees in breeding programs with a residual prediction deviation (RPD; [47]) above 2.3 (e.g., [48]). The RPD obtained for the *E. globulus* samples were 3.9, 3.8, 3.8 and 2.3 for Klason and Total lignin, S∶G ratio and Extractives, respectively.

DArT genotyping of all 303 individuals was carried out by Diversity Arrays Technology Pty Ltd (DArT P/L, Canberra, Australia) as described previously [36]. DArT dominant marker data were used to assess population structure, estimate pair-wise kinship coefficients and linkage disequilibrium, and perform association genetics analyses.

### Data analyses

A subset of 2,364 DArT markers was selected for the analysis based on quality parameters out of the 7,680 included on the genotyping array. The selected DArT markers had Call Rate (percentage of samples that could be scored as ‘0’ or ‘1’) greater than 80%, Reproducibility (reproducibility of scoring between replicated samples) greater than 97% and were polymorphic with frequencies of samples scored as ‘0’ or ‘1’ ranging between 0.95 and 0.05. Level of polymorphism for each marker was calculated by the expected heterozigosity: *H_e_* =  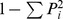 where *P_i_* is the frequency of the *i*th allele at the DArT marker locus. Because DArT markers are dominant, the allele frequencies were estimated making the frequency of ‘0’ genotypes equivalent to *P_i_* ^2^, assuming Hardy-Weinberg Equilibrium.

Genotypic data for a subset of 400 randomly taken DArT markers from the total of 2,364, were used to determine both population structure and pair-wise kinship coefficients among the 303 individuals, using the model-based Bayesian clustering algorithm implemented by STRUCTURE [12] and the kinship procedure of Hardy [13] using the software package SPAGeDi [49], respectively. STRUCTURE analyses were performed assuming an admixture model with default settings (i.e., no informative priors were used). STRUCTURE was run from 1 to 12 inferred clusters (*K*) with 10 independent runs for each *K*, each run starting with a burn-in period of 50,000 steps followed by 100,000 Markov Chain Monte Carlo iterations. The most probable value of *K* was selected according to the Δ*K* method [50]. Negative kinship values were set to zero following Yu *et al*. [11]. Pair-wise linkage disequilibrium (LD) between individual DArT markers was calculated by the square allele frequency correlation coefficient (r^2^) implemented in the program TASSEL version 3.0.137 [51] and their statistical significance was computed by 1,000 permutations using the two-sided Fisher's Exact test [52]. Mean r^2^ values were calculated separately for unlinked loci and for loci on the same chromosome. The 95^th^ percentile of the square root transforming of r^2^ distribution was taken as a population-specific critical value of r^2^ [53], beyond which LD was likely to be caused by genetic linkage.

The association mapping tests were carried out using two steps. In the first, data were standardized within each site and trait by subtracting each individual measurement from the mean trait value at that site and dividing the adjusted measurement by the site's standard deviation. The effect of large-scale environmental variation within each site was removed by exporting residuals from an analysis where replicate and incomplete block (for the Argentinian trial) was included as fixed or random effects, respectively. In the second step, these residuals were used as adjusted phenotype and single-marker associations were determined using the ***U***nified ***M***ixed-***M***odel [11]:
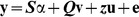
(Eq. 1)where **y**, **α**, **v**, **u**, and **e** are vectors of adjusted phenotypic observations, DArT effects (fixed), population effects (fixed), kinship effects (random), and residual effects, respectively, and ***S***, ***Q***, ***Z*** are incidence matrices relating **y** to **α**, **v**, and **u**, respectively. Several models were tested including *GLM* without population and family structures (GLM), and ***UMM*** with different combinations of population and family structures (Q, K, Q+K). Additionally, as recommended by Price *et al.* [54], the computationally demanding **Q** matrix was substituted by the principal coordinate's analysis' (PCA) **P** matrix resulting in two additional models (P and P+K). We removed the last column of the **Q** matrix to eliminate linear dependence between columns; however, all the columns (i.e., axes) were used in the **P** matrix to represent the population structure because it does not result in linear dependency. All analyses were conducted using TASSEL version 3.0.137 [51] and positive association were determined at the nominal *p*<0.05 level, and were further corrected for multiple testing using the false discovery rate (FDR) method for multiple comparisons [55] with *p*<0.05. The FDR thresholds were calculated using the QVALUE package [56] implemented in R (http://www.r-project.org/).

Model comparisons were based on the degree of deviation from uniform distribution and estimates of the mean square differences (MSD) between the observed and expected *p*-values of all DArT markers following the method described in Stich *et al.* [18]. A high MSD value indicates a strong deviation from a uniform distribution of *p*-values, suggesting that the type I error of the tested model could be substantially higher than the nominal α-level [18].

Pearson correlations were also calculated between the residuals from the measured traits using the CORR procedure in SAS [57]. Correlation between DBH and the wood chemical traits were very low (either positive or negative; i.e., from −0.06 to 0.03) and not significantly different from zero (*p*>0.27). However, wood chemical traits were highly inter-correlated (either positive or negative; i.e., from −0.43 to 0.96) and significantly different from zero (*p*<0.05), while PILO was only weakly positive correlated with DBH (0.16 *p* = 0. 05).

Based on the sequence information of the trait-associated DArT markers the following analyses were carried. Firstly, a sequence BLASTN search (*E*-value≤1e−3) against the *Eucalyptus grandis* genome database (version 1.1 available in Phytozome 6.0, http://www.phytozome.net/eucalyptus.php), was carried out. To assess the putative identities of these DArT markers, BLASTX searches were performed against the GenBank non-redundant protein database (http://blast.ncbi.nlm.nih.gov/Blast.cgi) with an *E*-value cut-off≤1e-10. Annotation and mapping routines were run with Blast2GO [58], using functional annotations and assigning gene ontology terms (GO terms) ([59]; http://www.geneontology.org/), and an enzyme classification number (EC number). Secondly, the Geneious pro 6.0.3 software (Biomatters http://www.geneious.com/) was used to assemble redundant DArT marker probe sequences, using the default parameter of 80% identity in a word length of 14 nucleotides. Thirdly, the genomic position the DArT marker probes as determined by aligning their sequences to the *Eucalyptus grandis* reference genome (version 1.1) [42], was used to scrutinize the gene content of the genomic window flanking the lignin S∶G ratio associated DArT marker using the Gbrowse tool of the *Eucalyptus grandis* genome version 1.1 available in Phytozome, (http://www.phytozome.net/eucalyptus.php).

## Results and Discussion

### DArT marker genotyping

**Figure 1:**
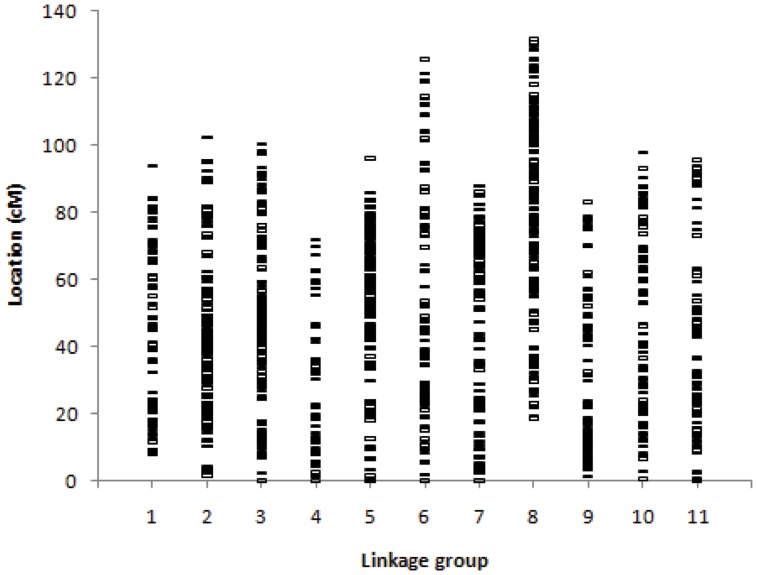
Reference genetic map position of 1,909 DArT markers assayed in the association study. Map position from Hudson *et al*. (2012).

DArT genotyping produced 2,364 high quality dominant markers with *H_e_* values ranging from 0.04 to 0.50 (the maximum value for a bi-allelic marker), with an average of 0.33 and most of them having *H_e_* values greater than 0.40 (Figure S1). Based on the *Eucalyptus* reference map [40], 1,909 of the 2,364 DArT markers had a known map location indicating that a reasonable genome-wide coverage from the recombination standpoint was provided by these markers (Figure 1). These 2,364 also provided, on average, a genome-wide physical genotyping density of one marker every ∼260 kbp or ∼0.5 cM based on recent genome-wide estimates of the relationship between physical distance and recombination frequency in the *Eucalyptus* genome [42]. An average of 174 markers per linkage group were assayed (1 marker/0.58 cM), with a minimum of 95 markers in linkage group 4 (1 marker/0.85 cM) and a maximum of 246 in linkage group 5 (1 marker/0.39 cM).

In the selected sample of 303 trees, pair-wise r^2^ estimates among the 1,909 DArT markers with known map position [40] varied from 0 to 1 with a mean of 0.09. The 95^th^ percentile of the distribution of unlinked r^2^ pairs was estimated at 0.025. However, the extent of LD within the different linkage group appeared variable. Mean pair-wise estimates of r^2^ across all the genome (i.e., intra- and inter-chromosomal) varied from 0.012 to 0.032. The number of pair-wise estimates r^2^ above the baseline 0.025 varied from 3.63% to 9.23%. However, a second-degree loess curve that fitted the r^2^ estimates did not reach the 95^th^ percentile baseline for any linkage group (Figure S2), indicating that the marker data obtained, although genome-wide, was not dense enough to detect consistent LD. This was expected, confirming that overall (i.e., using the intra- and inter-chromosomal and significant and non-significant r^2^ marker pairs) LD decays rapidly below a centimorgan, a recombination fraction found to correspond, on average, to 513 kbp [42]. The very few studies conducted in *Eucalyptus* species at the single gene level showed that LD decays rapidly within a few hundred base pairs [21], [60], [61], corroborating, as expected, that a higher marker density would be required to carry out a *bona fide* LD based genome-wide association study (GWAS).

### Population structure

**Figure 2:**
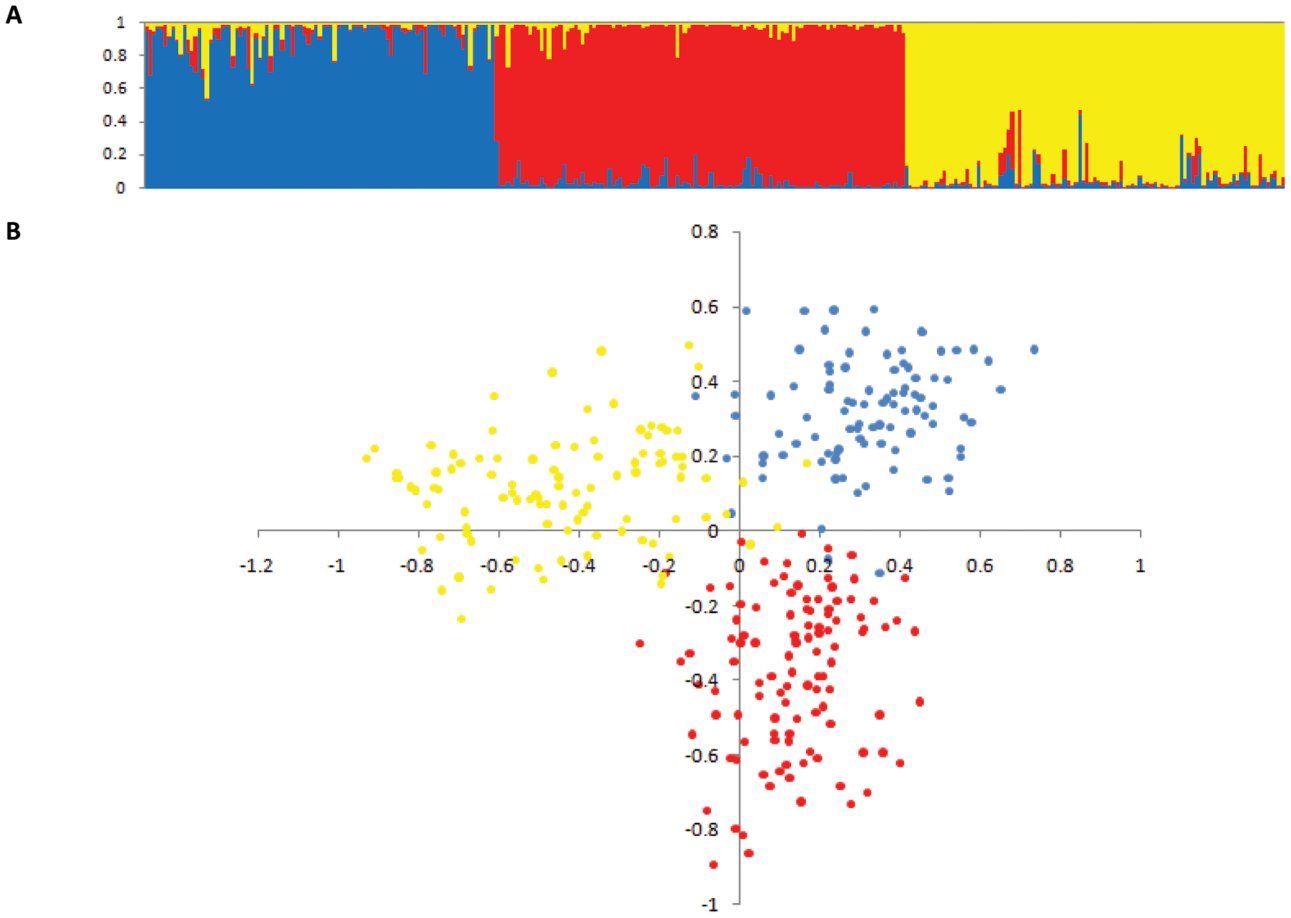
Results of population structure analyses using a Bayesian approach implemented by STRUCTURE (A) and PCA (Principal coordinate analysis) (B). Colors define three Genetic Group: Group 1 in blue (Eastern and Western Otways races and the Chilean provenance of the Uruguayan trials), Group 2 in red (Strzelecki Ranges race and *E. globulus* spp. *pseudoglobulus* (Naudin ex Maiden), and Group 3 in yellow (Furneaux, North-eastern and South-eastern Tasmania races, Chilean provenance of the Argentinean trial and Portuguese land race provenances).

The genetic structure obtained by STRUCTURE and PCA was similar (Figure 2). The first two principal components explained 24.84% (PC1) and 20.78% (PC2) of the variation in estimated genotypic state probabilities across the 400 DArT markers. The STRUCTURE analysis revealed three subpopulations, henceforth denoted as genetic groups (Figure 2A), which coincided with the broad geographical origin (i.e., races) in Australia [62]. Of the 303 trees, only four had membership probabilities set below 0.6 and had to be assigned to more than one genetic group. Genetic group 1 included 93 trees (31%), belonging to Eastern and Western Otways races (in Victoria, Australia), and to the Chilean provenance in the Uruguayan trials. Genetic group 2 included 109 trees (36%) belonging to the Jeeralangs (i.e., Strzelecki Ranges race in Victoria, Australia) and the *E. globulus* spp. *pseudoglobulu*s (Naudin ex Maiden). Genetic group 3 included 101 trees (33%) belonging to the Furneaux Group of Islands, and North-Eastern and South-Eastern Tasmanian races, the Chilean provenance in the Argentinian trial and the Portuguese land race. This clear racial genetic grouping is in line with the results reported in similar native population studies of *E. globulus* using microsatellite markers [63], [64]. They also coincide with Freeman *et al*. [65], in assigning the Portuguese land race to a Tasmanian origin. It is interesting to note however, that in the present study Jeeralang families were clustered together with *E. globulus* spp. *pseudoglobulu*s, whereas in Jones *et al*. [63], they were considered intergrades which were more closely related with Victorian *E. globulus* spp. *globulus* (such as Otways) and *E. globulus* spp. *bicostata*, than to *E. globulus* spp. *pseudoglobulu*s. The resulting patterns from the STRUCTURE program suggest that the inclusion of the **Q** or **P** matrices in the analyses is expected to affect the association test's statistical power (see below). In spite of the unambiguous clustering, consistent with the geographically and taxonomically distinct subpopulations, the overall *F*_ST_ value was only 0.095±0.01 indicating a moderate genetic differentiation between the three *E. globulus* genetic groups. The estimate is similar to Steane *et al*. [64] with 0.090±0.02, across an even wider range of core *E. globulus* and integrated races.

At the phenotypic level, visual inspection of the box plots based on the residual trait values in each of the three subgroups defined by STRUCTURE (Figure S3), and the *p*-values for differences of the least square means (Table S1) showed significant differences between some groups in all traits studied, except Extractives. Victorian races (Genetic Group 1 and 2) had superior DBH, but also higher Klason and total lignin and Extractive content, compared with Tasmanian races (Genetic Group 3), hence indicating better growth but poorer pulp quality attributes. As expected, the Jeeralang and *E. globulus* spp. *pseudoglobulu*s material (Genetic Group 2) had the highest density (lowest Pilodyn) of the three [46], [62].

### Family structure

**Figure 3:**
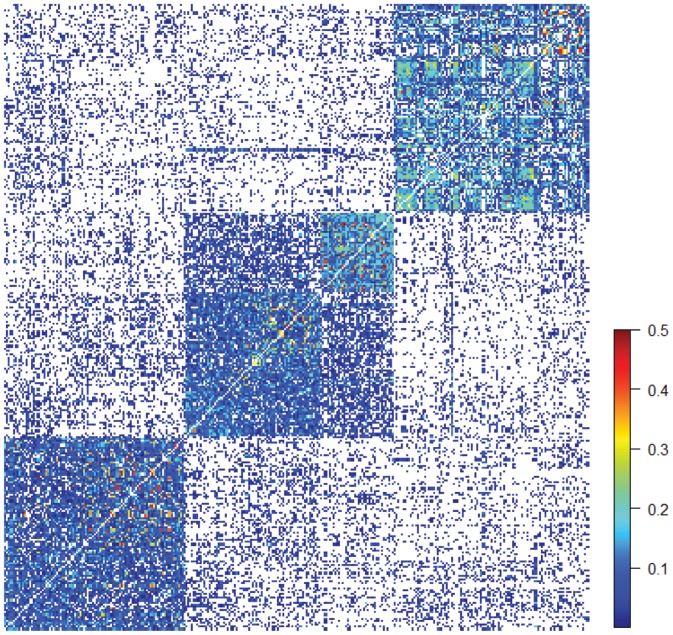
Heat map of the pair-wise relationship coefficients among the 303 *E. globulus* trees produced from the SPAGeDi program showing the three Genetic Groups: Genetic Group 1, 2 and 3 from right to left. The heat scale represents pair-wise relationship coefficients for all pairs of individual trees. Values greater than 0.5 are not shown and account for only 0.08% of the distribution.

The pair-wise relatedness among the 303 trees resulted in the identification of an array of variable relationships (Figure 3), indicating the presence of a within population familial structure, and providing justification to include the **K** matrix in the association genetics analyses. We knew *a priori* that some of the trees were related, because they belonged to the same open-pollinated family, the average relationship coefficient for these trees was 0.232 (closest to the pedigree expected value of 0.25), whereas the average for the unrelated pairs of trees was 0.032 (closest to the expected pedigree value of zero). Mean relationship within genetic groups was 0.076, 0.069 and 0.083 for genetic groups 1, 2, and 3, respectively. As expected, the relationship among trees from the same genetic group was greater than those from different genetic groups. This was particularly true between groups 2 and 3 (0.011). The most frequent class of genetic relationship was for values between 0.00 – 0.05 (75.6%), followed by values between 0.05 – 0.10 (12.7%) (Figure S4). Given that the family structure is truly present, then correlation between pair-wise relatedness and pair-wise geographic distance should be negative and significant. In fact, negative and highly significant Mantel correlation was observed for the DArT markers (−0.49, *P*<0.0001), confirming the presence of isolation-by-distance as the probable cause of the family structure found and once again justifying the inclusion of the **K** matrix in the association genetics analyses.

### Association genetics models comparison

**Figure 4:**
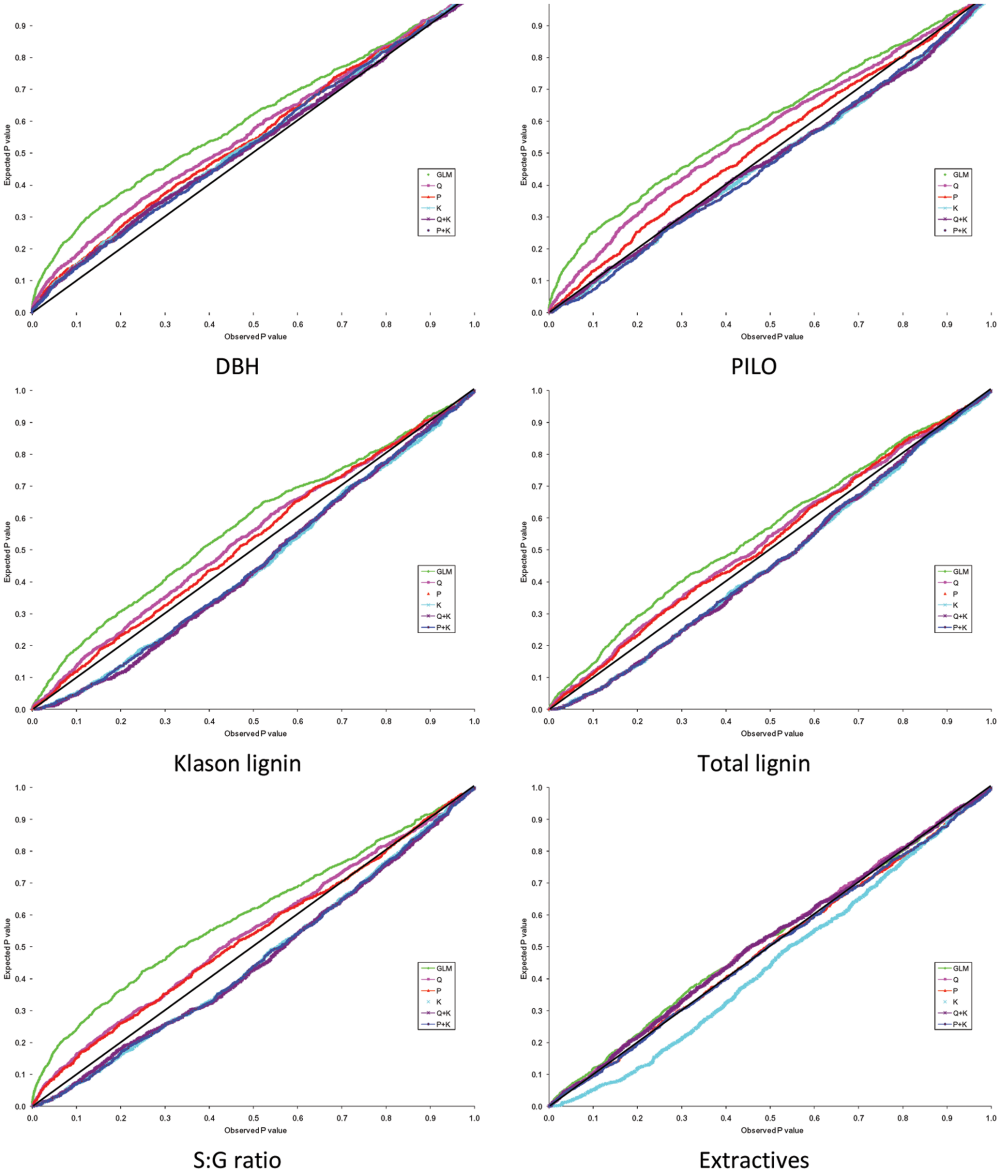
Plots of the observed vs. expected *p-values* for the six association genetics models studied for all traits. See text for models' and traits' abbreviation.

**Figure 5:**
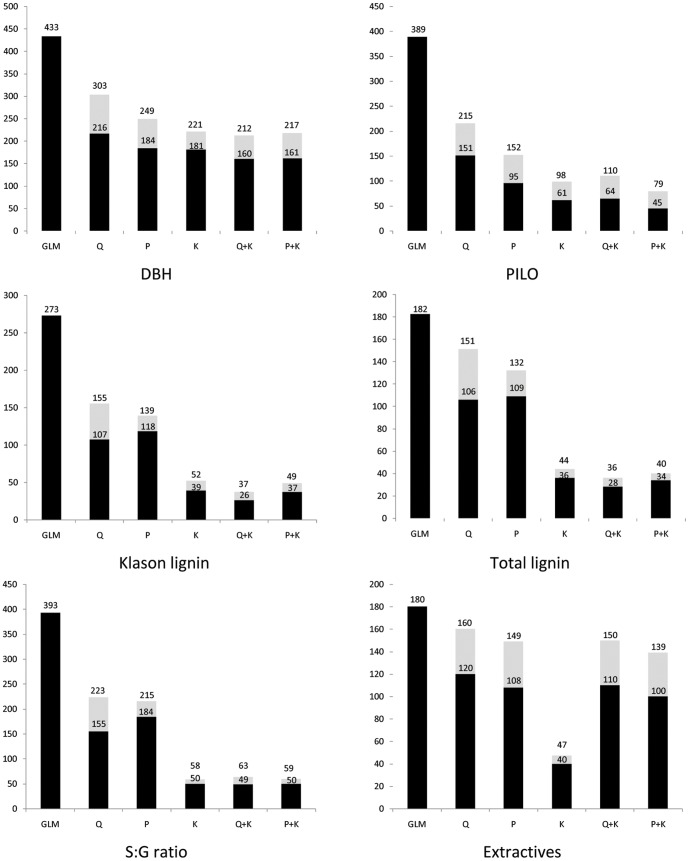
Association mapping results from the different linear models indicating the number of significant (*p*-value≤0.05) DArT markers associated with growth and wood properties traits. In each bar, the gray and black areas represent the number of significant DArT markers from the specific model (i.e., GLM, Q, P, K, Q+K and P+K) and overlapping between the specific model and GLM, respectively. See text for models' and traits' abbreviation.

Results from association analyses clearly indicated that there was no universal/single model that suited all the studied traits (Table 2, Figure 4). Overall, the GLM did not perform well. The mean square difference (MSD) between observed and expected *p*-values was highest for the GLM model (Table 2) and the distribution for MSD was substantially deviated from uniformity (Figure 4). It is noteworthy to note the drastic difference when population structure was included as a source of variation in the model. Irrespective of which population structure matrix was used (**Q** or **P**), the models that included a population structure performed consistently better than those that did not for all traits (Table 2, Figure 4); this was expected given the geographical structure among the *E. globulus* population being studied. The MSD of the **Q** model (and **P** model) ranged from 0.000469 (0.000069) for Extractives to 0.006154 (0.001328) for PILO, and was considerably lower than the value found for the GLM model (Table 2). Additionally, the curves in Figure 4 show that accounting for population structure (**P** or **Q**) dramatically increases the statistical power. Moreover, the GLM model produced a number of apparent false positive or spurious associations across all traits (Figure 4). As a result, using the **Q** or **P** matrix resulted in a considerable reduction in the number of significant marker-trait associations before multiple testing corrections were applied (*p*<0.05; see Figure 5 for a comparison between the simplest model (GLM) and both, the Q or P models). The importance of accounting for structure in the analysis was recently demonstrated in *E. globulus* [66]. In that study, Külheim *et al*. [66] detected hundreds of false-positive associations demonstrating the pitfall associated with ignoring geographical origin.

**Table 2:** Mean squared differences (MSD) between observed and expected *p*-values for the six association genetics models applied in the study.

	Trait					
Model	DBH	PILO	S∶G ratio	Klason lignin	Total lignin	Extractives
**GLM**	0.013445	0.012289	0.013029	0.007098	0.004264	0.000624
**Q**	0.004725	0.006154	0.002134	0.001836	0.001214	0.000469
**P**	0.002502	0.001328	**0.001497**	**0.000866**	**0.000907**	**0.000069**
**K**	0.001250	0.000800	0.002208	0.002828	0.001893	0.003370
**Q+K**	0.001044	**0.000718**	0.002461	0.003167	0.001918	0.000427
**P+K**	**0.001017**	0.000798	0.002293	0.002508	0.001725	0.000070

The selected models are highlighted in bold (see text for models' and traits' abbreviations).

In the present study, we have incorporated the kinship relationship among trees. Accounting for family structure is expected to remove most of the variance associated with close relatedness that would not have been explained by the **Q** or **P** matrices [16]. In our study, the inclusion of the **K** matrix in the model appeared to be trait dependent. Although 76% of pair-wise relationship estimates were close to 0, for DBH and PILO the K model outperformed the Q or P models, i.e. produced smaller MSD values (Table 2) and showed a similar expected distribution of *p*-values (Figure 4), whereas the opposite was true for the wood chemical traits (S∶G ratio, Klason lignin, Total lignin, and Extractives). Furthermore, the K model produced a more stringent reduction in the number of significant markers (*p*<0.05) with respect to the Q and P models (from 1.18% to 6.98%) in the six traits tested (Figure 5). This finding suggests that, when there are complex interrelationships among trees, both within and among subpopulations, fitting a Q (or P) model may not be adequate to reduce the number of spurious associations. Such results highlight the importance of accounting for the various sources of variation due to structure in the model (i.e., **Q** and/or **K** matrices). Yu *et al*. [11] presented a compelling scenario where, in some cases, population structure was not needed as family structure was adequate to capture the underlying structure in the data (e.g., ear height). Generally, family structure captures substantial amounts of variation caused by population structure and population structure inclusion would be only necessary in cases where obvious regional differences exist.

As expected, the complete Q+K [11] and P+K [54] models were equivalent, i.e. the MSD values between observed and expected *p-*values were similar and have similar MSD distributions (Figure 4). Furthermore, both models (i.e., Q+K and P+K) yielded similar reduction of number of significant (*p*<0.05) marker-trait associations for all studied traits (Figure 5). These findings demonstrate that association mapping models based on the two population structure matrices **Q** and **P** would be equally appropriate, when the **K** matrix is included. This indicates that it may be possible to replace the computationally intensive STRUCTURE algorithm with a simple PCA, an important result when large number of markers and large population sizes are included in the ***UMM***, an increasingly common situation as much higher throughput sequencing-based SNP genotyping systems are becoming accessible for forest trees [67], [68], [69]. Similar conclusions have been reached by Zhao *et al*. [70] in soft winter wheat (*Triticum aestivum* L.) and by Stich *et al*. [18] in *Arabidopsis thaliana*.

Based on the MSD values (Table 2), the distribution of the expected and observed *p*-values (Figure 4), and the number of significant marker-trait associations (Figure 5), the best models by trait combinations used for comparing association results were as follows: P+K model for DBH, Q+K model for PILO, and P model for all other wood chemical traits (S∶G ratio, Klason lignin, Total lignin, and Extractives).

### DArT marker-trait associations

A total of 14,184 association tests were performed (2,364 DArT markers versus six traits). This resulted in 962 positive associations before multiple testing corrections at the significance level of *p*<0.05. Using 1,000 permutations in an *F*-statistic test for correcting the rate of false discoveries, the number of positive single marker-trait associations was reduced to 18 (16 for DBH and 2 for S∶G ratio; Table 3). These results could apparently suggest that wood specific gravity (PILO) and possibly the other chemical properties of wood (S∶G ratio, Klason lignin, Total lignin, and Extractives) may involve fewer genes, resulting in a lower number of DArT markers associations, at least compared with growth (DBH), which is expected to be a more complex trait influenced by many genes. In fact, chemical wood properties often involve a single biosynthetic pathway and consequently one could expect a lower number of marker-trait associations when compared to complex traits such as growth or wood density that are affected by many different physiological processes. However, this is not what has been emerged in more recent and better powered biparental QTL studies in *Eucalyptus*. For example, Gion *et al*. [71] detected similar number of QTLs for growth and wood chemical traits in *E. urophylla* and *E. grandis*. Freeman *et al*. [44] identified a lower number of QTLs for DBH (11) than for wood chemical traits (average 14.2 QTLs/trait). Moreover, in Resende *et al.* [35], the number of markers with largest effect that maximized the proportion of heritability captured by Genomic Selection for pulp yield was essentially the same (∼200) as the number that did so for height growth. These results suggest that for any given experimental population size and genetic background there are other issues determining the likelihood of QTL detection such as the extent of phenotypic variance and the heritability of the trait.

**Table 3:** Association mapping results from the different linear models indicating the number (and percentage) of significant markers found associated with growth and wood property traits after correction for multiple testing (FDR *p*-value≤0.05).

	Trait					
Model	DBH	PILO	S∶G ratio	Klason lignin	Total lignin	Extractives
**GLM**	126 (5.3)	71 (3.0)	45(1.9)	16(0.7)	0 (0.0)	0 (0.0)
**Q**	44 (1.9)	5 (0.2)	0 (0.0)	0 (0.0)	0 (0.0)	0 (0.0)
**P**	28 (1.2)	0 (0.0)	**2 (0.1)**	**0 (0.0)**	**0 (0.0)**	**0 (0.0)**
**K**	18 (0.8)	0 (0.0)	0 (0.0)	0 (0.0)	0 (0.0)	0 (0.0)
**Q+K**	21 (0.9)	**0 (0.0)**	0 (0.0)	0 (0.0)	0 (0.0)	0 (0.0)
**P+K**	**16 (0.7)**	0 (0.0)	0 (0.0)	0 (0.0)	0 (0.0)	0 (0.0)

In bold, the number of significant marker-trait associations detected in the selected models by trait combination (see text for models' and traits' abbreviations).

The amount of phenotypic variation explained by each marker (R^2^) in the selected models was always modest, accounting for 4.02% to 13.76% (only three of them had major effects above 10%, Table 4), with an average of 7.27% for DBH and 5.64 for S∶G ratio. These small R^2^ values are consistent with the genetic architecture of complex traits expected to be influenced by many loci, each with a relatively small effects (i.e., Fisher's 1918 infinitesimal model). Recent studies in *Eucalyptus* have reported low numbers of QTLs for growth and wood quality traits, whose effects rarely accounted for more than 5% of the observed variance [43], [44], [71], [72], [73], [74], [75]. In *E. globulus*, Bundock *et al*. [72] detected six QTLs for diameter (DBH) and height and two for PILO explaining between 7.2% and 10.1% of the phenotypic variation. Freeman *et al*. [73] identified 11 QTLs for growth and wood properties traits, with each QTL explaining between 3.8% and 12.3% of the phenotypic variation. In *E. nitens*, Thumma *et al*. [75] found 36 QTLs for Klason and total lignin, extractives and density amongst other wood traits, with R^2^ varying from 2.8% to 7.3% and averaging 5%. Gion *et al*. [71] detected 117 QTLs for a number of wood properties (including chemical, technological, physical, mechanical and anatomical) and growth traits in an interspecific cross between *E. urophylla* and *E. grandis*. In conclusion, most QTLs had effects below 5% and only 13 of them had effects above 15%. However, for growth traits the R^2^ values varied from 4.1% to 42.2% and from 5.0% to 37.0% for wood traits. In four-year-old progenies of two interspecific backcross families of *E. urophylla* and *E. grandis*, Kullan *et al*. [43] detected 5 QTLs for DBH and 12 for wood basic density. In this case, the R^2^ varied from 4.6% to 8.0% averaging 5.9% for DBH, and from 3.1% to 12.2% averaging 5.9% for wood density.

**Table 4:** Significant DArT marker-trait associations for DBH and S∶G ratio retained for the six models.

Model/Trait	Marker name	GLM			Q			P			K			Q+K			P+K		
		*p*-value	*Q*-value	R^2^	*p*-value	*Q*-value	R^2^	*p*-value	*Q*-value	R^2^	*p*-value	*Q*-value	R^2^	*p*-value	*Q*-value	R^2^	*p*-value	*Q*-value	R^2^
**DBH**	ePt-503280	1.5E-07	1.9E-05	9.1%	1.0E-05	1.7E-03	6.1%	1.9E-04	2.2E-02	4.1%	5.1E-05	8.9E-03	6.4%	7.3E-05	1.6E-02	6.1%	**3.1E-04**	**4.8E-02**	**4.3%**
**DBH**	ePt-503742	2.2E-08	3.5E-06	10.4%	1.4E-05	2.2E-03	6.0%	3.7E-05	8.0E-03	5.0%	4.8E-05	8.9E-03	6.5%	1.1E-04	1.9E-02	5.8%	**1.6E-04**	**3.2E-02**	**4.7%**
**DBH**	ePt-563549	1.1E-04	5.5E-03	5.0%	8.3E-05	7.7E-03	4.9%	1.0E-04	1.4E-02	4.4%	1.7E-04	2.2E-02	5.7%	2.8E-04	3.6E-02	5.2%	**3.8E-04**	**5.0E-02**	**4.3%**
**DBH**	ePt-572035	6.2E-16	3.1E-13	20.8%	3.5E-12	1.6E-09	14.7%	1.7E-09	8.0E-07	10.3%	1.3E-08	6.5E-06	14.6%	3.1E-08	1.7E-05	13.5%	**3.1E-07**	**1.6E-04**	**9.8%**
**DBH**	ePt-572842	5.2E-15	1.5E-12	19.6%	4.7E-11	1.7E-08	13.2%	1.6E-08	5.4E-06	9.3%	3.6E-08	1.3E-05	12.6%	6.1E-08	2.6E-05	12.0%	**4.6E-07**	**1.8E-04**	**8.8%**
**DBH**	ePt-574221	5.2E-15	1.5E-12	20.7%	5.9E-11	1.8E-08	14.0%	1.7E-08	5.4E-06	9.6%	3.7E-08	1.3E-05	13.9%	7.9E-08	2.8E-05	13.0%	**1.3E-06**	**4.3E-04**	**9.0%**
**DBH**	ePt-574487	8.4E-06	7.8E-04	7.3%	1.1E-04	9.3E-03	5.1%	1.0E-04	1.4E-02	4.8%	7.2E-05	1.1E-02	6.7%	1.8E-04	2.7E-02	5.8%	**2.2E-04**	**4.0E-02**	**4.8%**
**DBH**	ePt-575116	2.6E-06	2.8E-04	7.6%	2.6E-05	3.4E-03	5.6%	2.1E-04	2.4E-02	4.1%	3.3E-04	3.8E-02	5.7%	4.0E-04	4.5E-02	5.5%	**2.4E-04**	**4.0E-02**	**5.0%**
**DBH**	ePt-599304	7.0E-16	3.1E-13	20.7%	2.1E-12	1.3E-09	15.0%	8.9E-11	5.7E-08	12.0%	3.2E-09	2.2E-06	14.5%	3.4E-09	2.5E-06	14.2%	**1.5E-08**	**1.0E-05**	**11.1%**
**DBH**	ePt-638303	8.5E-04	1.9E-02	3.7%	3.3E-04	2.2E-02	4.0%	2.8E-04	3.1E-02	3.8%	2.3E-03	1.3E-01	3.5%	1.9E-03	1.3E-01	3.6%	**3.9E-04**	**5.0E-02**	**4.0%**
**DBH**	ePt-639597	1.5E-06	1.7E-04	7.9%	7.2E-06	1.3E-03	6.4%	1.3E-04	1.7E-02	4.4%	1.1E-04	1.6E-02	6.1%	9.9E-05	1.9E-02	6.1%	**9.6E-05**	**2.2E-02**	**5.2%**
**DBH**	ePt-640845	4.9E-10	9.5E-08	13.9%	4.2E-08	9.8E-06	10.1%	7.3E-05	1.4E-02	4.9%	5.0E-06	1.3E-03	10.0%	1.2E-05	2.9E-03	9.0%	**3.4E-04**	**4.9E-02**	**5.1%**
**DBH**	ePt-641597	1.1E-19	9.8E-17	25.8%	8.7E-16	8.1E-13	19.3%	2.0E-12	2.0E-09	14.1%	1.2E-10	1.3E-07	18.6%	1.9E-10	2.1E-07	17.9%	**1.3E-09**	**1.3E-06**	**13.7%**
**DBH**	ePt-641619	9.9E-12	2.2E-09	14.8%	1.0E-07	2.1E-05	8.6%	6.7E-06	1.6E-03	5.9%	6.9E-06	1.6E-03	8.1%	1.2E-05	2.9E-03	7.5%	**7.4E-05**	**1.9E-02**	**5.2%**
**DBH**	ePt-643627	4.9E-14	1.2E-11	18.9%	6.6E-10	1.8E-07	12.1%	2.5E-07	6.8E-05	8.0%	2.2E-07	6.5E-05	11.8%	4.9E-07	1.5E-04	10.9%	**5.1E-06**	**1.5E-03**	**7.6%**
**DBH**	ePt-644292	4.2E-21	7.4E-18	26.3%	1.7E-16	3.1E-13	19.1%	1.1E-13	2.2E-10	14.9%	1.3E-11	2.7E-08	18.7%	2.0E-11	4.3E-08	18.1%	**2.8E-10**	**5.6E-07**	**13.6%**
**S∶G ratio**	ePt-503848	4.7E-06	1.0E-03	7.2%	7.8E-05	8.3E-02	4.9%	3.6E-05	3.9E-02	5.4%	2.2E-03	1.0E+00	4.4%	3.5E-03	1.0E+00	3.6%	3.1E-03	1.0E+00	4.1%
**S∶G ratio**	ePt-638347	2.8E-06	7.2E-04	7.8%	6.8E-05	8.3E-02	5.2%	2.9E-05	3.9E-02	5.8%	1.3E-03	1.0E+00	5.1%	2.3E-03	1.0E+00	4.1%	1.8E-03	1.0E+00	4.7%

Shown are *p*-values after correction for multiple testing (FDR *p*-value≤0.05, *Q*-value) and estimates of percent phenotypic variance putatively explained (R^2^). Highlighted in bold, the best trait by model combinations reported based on the degree of deviation from the uniform distribution and estimates of the mean square differences (MSD) between the observed and expected *p*-values (see text for models' and traits' abbreviations).

As expected, we did not find any overlap between the associations found for DBH and S∶G ratio, consistent with the lack of phenotypic correlation between them (Pearson coefficient -0.053 *p*-value 0.37). However, it is interesting to note that the magnitude of these 18 associations did not change across the six models (*p-values*<0.05 and FDR *p*-value≤0.05), except for marker ePt-638303 for DBH which was not significantly associated when using the K and Q+K models. Such consistency of results, notwithstanding the slight differences in R^2^ estimates across models, is a promising indication that the associations are indeed real (Table 4). Nevertheless, given the limited size mapping panel, these marker-trait associations will require further validation in independent populations.

It is important to highlight, however, that while the genomic locations of the DArT-marker-trait associations found in this study should be informative, we acknowledge the fact that the estimated magnitudes of effects are likely upwardly biased. This is probably the case for most marker-trait associations and QTLs mapped so far in forest trees, although this is rarely recognized. Overestimation of the proportion of variance explained in association studies is a phenomenon that has traditionally been called the ‘winner’s curse' [76] or the ‘Beavis effect’ [77] when referring to QTLs mapped in limited size biparental crosses. Reasons for this include the usually limited sample sizes used and the fact that a single dataset is used for both discovery and parameter estimation, causing a correlation between the test statistic and the estimated marker effect size. Additionally genome-wide associations and QTLs are typically estimated ignoring the rest of the genome which also contributes to an upward bias. This problem can be mitigated by fitting all markers simultaneously as random effects, an approach that has been proposed and adopted for Genomic Selection [78] or improved ways of carrying out GWAS [79]. This approach, successfully applied in several animal and plant species, has been shown to result in a much larger number of effects detected (hundreds), with considerably smaller magnitudes (<1%), converging to a quasi-infinitesimal model while explaining very large proportions of the heritability for complex traits [80]. Recently three Genomic Selection studies were carried out in large populations (>700-900 individuals) of *Pinus* and *Eucalyptus*, all of them showing that hundreds of small marker effects distributed across the whole genome contribute to height growth and wood quality traits [34], [35], [81], in clear contrast to the long held beliefs from QTL mapping experiments of the existence of at least a few loci with large effects (5-15%) controlling such complex traits.

We compared the results of estimating markers effects simultaneously as random effects or fixed effects using Eq. 1. DArT markers effects were fitted as random using Bayesian least absolute shrinkage and selection operator (LASSO) in the BLR package for R, version 2.12.2 ([82], [83]). Results indicated that the estimated absolute effects of the eighteen significant DArT marker-trait associations (*Q*-value<0.05) under a fixed model (Eq. 1) were 21 and 24 times larger than those estimated under the random model (Bayesian LASSO) for DBH and S∶G ratio, respectively. Furthermore, the proportion of the genetic variance explained by the markers (2 *p_i_* (1-*p_i_*) 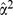; where *p_i_* is the frequency of one allele at that locus and 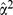 is the estimated marker effect) decrease on average from 19.64% to 0.06% for DBH and from 11.78% to 0.02% for S∶G ratio, when estimated as fixed and random effects, respectively. Increasing the size of the association mapping populations is expected to reduce these differences.

### Genomic locations and putative annotations of marker-trait associations

The present study identified 18 significant DArT marker-trait associations after correcting for multiple testing and accounting for racial and family structure in the data. Fifteen of these 18 DArT marker probes had been sequenced, 13 of them were physically aligned to a unique position on the 11 chromosome scaffolds [42], and two were aligned to still unanchored small scaffolds (numbered beyond 11) (Table 5). The 15 DArT probes were assembled in 13 non redundant sequences (two contigs and 11 singletons) consistent with their alignment position reported earlier [40]. Markers ePt-575116 and ePt-639597, associated with DBH belong to the same locus located at 28,708,000 bp and markers ePt-503848 and ePt-638347, associated with S∶G ratio, map to position 30,915,259 bp (Table 5). Annotation of the 15 sequences was initially based on primary sequence homology searches. Under an *E*-value threshold of <1.e^-10^, eight DArT marker sequences, all associated with DBH, had significant BLASTX matches (Table 5) while no match to genes was found for the two DArT markers associated with S∶G ratio. Sequences with a positive BLASTX match were annotated using GO terms. As a result, GO terms were assigned to eight sequences totalizing 28 GO terms (Table 5). Blast2GO analysis at process level 3 showed that among the different biological processes (18 GO terms), the sequences belong to ‘Macromolecule Metabolic Processes’ (4), ‘Primary Metabolic Process’ (4), ‘Nitrogen compound metabolic process’ (3), ‘Cellular metabolic process’ (3), ‘Biosynthetic process’ (1), ‘Establishment of localization’ (1), and most surprisingly, ‘Response to abiotic stimulus’ (1), ‘Response to stress’ (1). Of these sequences, two were assigned with EC numbers: Serine endopeptidase (EC: 3.4.21.0) and RNA-directed DNA polymerase (EC: 2.7.7.49).

**Table 5:** Genomic position of the DArT marker-trait associations detected and putative underlying genes found by a simple BLASTX search in the *Eucalyptus grandis* reference genome version 1.1 (available in Phytozone) (see text for traits' abbreviations).

Trait	DArT Marker probe	SequenceLength (bp)	Start position (bp)*^a^*	Chromosome scaffold^b^	Sequence Description	E-value	GO Terms^c^	EC number^d^
**DBH**	ePt-638303	1489	21,098,595	1	nucleotide binding	6.53E-19	C:endomembrane system; F:nucleotide binding; P:biological_process	
**DBH**	ePt-575116	340	28,708,000	1				
**DBH**	ePt-639597	425	28,707,914	1				
**DBH**	ePt-572842	646	33,519,455	1	hypothetical protein VITISV_027476 [*Vitis vinifera*]	3.99E-80	F:metal ion binding; F:nucleic acid binding	
**DBH**	ePt-643627	584	38,832,055	3	retrotransposon gag protein	3.05E-64	F:RNA-directed DNA polymerase activity; F:DNA binding; P:RNA-dependent DNA replication; F:RNA binding; F:zinc ion binding; P:DNA integration	EC:2.7.7.49
**DBH**	ePt-563549	516	71,570,031	3	membrane-bound transcription factor site-1 protease	1.01E-14	P:hyperosmotic salinity response; F:serine-type endopeptidase activity; C:Golgi apparatus; P:proteolysis; C:endoplasmic reticulum	EC:3.4.21.0
**DBH**	ePt-503742	371	70,057,987	5				
**DBH**	ePt-641619	316	7,960,919	6	disease resistance protein	3.64E-25	F:inorganic cation transmembrane transporter activity; F:metal ion binding; F:nucleic acid binding; P:transport; P:cellular process; C:membrane	
**DBH**	ePt-503280	689	14,534,768	6	bed finger-nbs-lrr resistance protein	3.54E-44	F:binding	
**DBH**	ePt-640845	717	48,412,568	7	hypothetical protein VITISV_041092 [*Vitis vinifera*]	2.64E-14	F:DNA binding; F:zinc ion binding; P:DNA integration	
**DBH**	ePt-574221	663	369,118	10				
**DBH**	ePt-644292	215	41,307	411	retrotransposon gag protein	4.10E-24	F:DNA binding; P:DNA integration	
**DBH**	ePt-599304	291	8,417	1,553				
**DBH**	ePt-572035	n.a.	n.a.	n.a.				
**DBH**	ePt-574487	n.a.	n.a.	n.a.				
**DBH**	ePt-641597	n.a.	n.a.	n.a.				
**S∶G ratio**	ePt-503848	635	30,915,259	10				
**S∶G ratio**	ePt-638347	646	30,915,259	10				

**NOTE**: *^a^* Burrows-Wheeler Alignment position of DArT marker probe to the *Eucalyptus grandis* reference genome as described in [42].

*^b^* *Eucalyptus grandis* genome chromosome scaffold to which DArT marker probe had best alignment score as described in [42]; scaffold numbered over 11 correspond to still unanchored sequence scaffolds.

*^c^*P, biological process; F, molecular function; C, cellular component.

*^d^*EC number, enzyme classification number.

n.a. sequence not available for DArT marker probe.

Comparison of genomic position of the associations found with previous QTL mapping studies were carried out, although to date, few have been the QTL detection reports in *Eucalyptus* using DArT markers. Interestingly, however, one of the DArT markers (ePt-503848) associated with S∶G ratio in our population was also mapped to a QTL by Freeman *et al*. [44] for the same trait on linkage group 10. QTLs for lignin composition related traits were also mapped to the same segment of linkage group 10 early on by Thamarus *et al*. [84] for pulp yield (a trait strongly correlated with S∶G ratio), by Thumma *et al*. [75] also for pulp yield, and by Gion *et al*. [71] for Klason lignin. The strong concurrence between independent reports mapping a QTL for lignin composition traits on this region of linkage group 10 prompted us to scrutinize the corresponding genomic regions in the vicinity of our DArT marker ePt-503848, associated with S∶G ratio. This marker was positioned starting at 30,915,259 bp on chromosome scaffold 10 which has approximately 40 Mbp total size. The analysis revealed the presence of the ferulate 5-hydroxylase (F5H) gene, annotated with strong support at position 29,822,332 bp of the same chromosome 10. Ferulate 5-hydroxylase (F5H), also referred to coniferaldehyde 5-hydroxylase is a key enzyme involved in synthesizing the monolignol sinapyl alcohol and, ultimately, S lignin moieties. F5H therefore affects the partitioning between the two major monolignols, coniferyl and sinapyl alcohols. The major role of this gene in controlling the S∶G ratio was further shown by the drastic increase in the Syringyl monomer levels when overexpressed in transgenic poplars, as reported by different research groups [85], [86], [87]. The distance between DArT marker ePt-503848 and the F5H gene is slightly over 1 Mbp. However recent comparative mapping analyses showed that the considerable genome size difference between the *E. grandis* (640 Mbp) and *E. globulus* (545 Mbp) genomes is largely due to the sum of many small insertions/deletions widely distributed across the genome (Josquin Tibbits unpublished). It is therefore reasonable to speculate that this 1 Mbp difference between the marker and the gene could in fact be much smaller in the *E. globulus* genome. As tentative as it may be, this putative co-localization provides an appealing indirect biological validation of the association we found for S∶G ratio in our study.

Additional comparisons between the associations found in our study and QTLs mapped for diameter growth in previous reports are only possible at the coarse linkage group level. For example, as in our study, QTLs for diameter growth were also detected on linkage groups 3, 5, 7 and 10, in a recent multi-pedigree *E. globulus* [44] report. If we consider, however, the genome-wide results reported by Resende *et al.* [35] in two large hybrid *Eucalyptus* breeding populations with 780 and 920 individuals respectively, several hundred DArT markers associated with DBH were found spread out across all 11 chromosomes. When fitted in genomic selection models, the 200 associated DArT markers of largest effect captured over 80% of the heritability for diameter growth (DBH), although the individual effects of these markers rarely surpassed 1%. Assuming that this is the most likely genetic architecture of this growth trait in *Eucalyptus*, and therefore that the whole genome takes part in controlling this complex trait, comparative QTL position analyses for such multifactorial traits become too uncertain to be of any use.

## Conclusions

The results of this study highlight the importance of taking into account all relevant sources of variation and assessing the relative value of using different analytical models in association mapping studies in forest trees. The approach taken in our analysis was especially rigorous to avoid declaring false marker-trait associations, generally considered more detrimental than false negatives in genome-wide associations studies. In particular, it was important to account for possible genetic structure in the data, both from regional genetic groups (races and provenances) and within population kinship relationships (cryptic family presence). It was also essential to use more stringent significant threshold levels in the tests, such as 1 or 5%, and to look at the mean squared differences between observed vs. expected *p*-values between the different models, when a considerable trait by model interaction was observed. Assessing the impact of different population structures and analytical models on the power and accuracy of association genetics in other *Eucalyptus* populations and forest tree species in general will be important to validate our results. On other hand, had phenotype prediction by a Genomic Selection approach been the goal, the converse would be true. GS estimates all marker effects simultaneously, precluding the prior search for significant associations by rigorous significance tests, but rather retaining all markers or subsets of them as predictors of performance, while focusing exclusively on selection efficiency. In such a context, most likely the several hundred associations we had found prior to stringent correction for false discoveries would be retained in a predictive model, a subject we are currently investigating, in line with recent findings in *Eucalyptus* [35].

Our estimates of LD corroborated our expectation that a much higher marker density would be required to carry out a genuine association genetics study in this population, although this is by far the densest genotyping coverage used specifically for association mapping in *Eucalyptus* to date. Moreover, given the size limitation of our mapping panel we recognize that we had limited power to detect many smaller effects, and that the marker-trait associations found, despite the high statistical stringency used to declare them, will require independent validation. This is likely the current status of most association mapping studies previously reported in *Eucalyptus* that used even smaller population sizes, varying from 86 to 323 and averaging 186 (reviewed by Grattapaglia *et al*. [88]), exception made by recent Genomic Selection experiments [35]. Nevertheless, unlike candidate gene based association studies that are inherently biased by the initial choice of what genes to assay, this association study, although carried out at a marker density far from ideal, points, in an unbiased way, to genomic regions putatively involved in controlling growth in general and S∶G ratio in particular. While several independent, although indirect, pieces of evidence were compiled from the literature pointing to the F5H gene as a candidate underlying the single marker-trait association found for S∶G ratio, in the case of volume growth the picture is certainly blurrier. As tempting as it may be to propose genes identified as candidates for controlling volume growth, given the paramount complexity of this trait, considerably more experimental evidence and independent validation would be required to do so. Additionally, from the applied standpoint, simply monitoring the inheritance of a handful of genes would hardly impact the advancement of a breeding population.

## Supporting Information

Figure S1 **Distribution of expected heterozigosity (** ***H_e_*** **) values for the 2,643 DArT markers genotyped in the** ***E. globulus*** **mapping population (average** ***H_e_*** **= 0.33).**
(DOCX)

Figure S2 **Linkage Disequilibrium (LD) decay as measured by r^2^ for pairs of DArT markers across the genome (i.e., intra- and inter-chromosomal), against genetic distance (in cM) in the 303** ***E. globulus*** **trees.** The second-degree Loess fitting curve illustrates the LD decay based on the nonlinear regression of r^2^ on genetic distance while the horizontal line indicate the baseline r^2^ values based on the 95^th^ percentile of the r^2^ values distribution.
(DOCX)

Figure S3 **Boxplots of growth and wood properties variation in the studied sub-populations.** See text for traits' abbreviation.
(DOCX)

Figure S4 **Distribution of pair-wise relatedness coefficients among the 303** ***E. globulus*** **trees.** Values greater than 0.5 are not shown and account for only 0.08% of the distribution.
(DOCX)

Table S1 Significances for the differences of the least square means based on subpopulations estimated by the STRUCTURE software. See text for traits' abbreviation.
(DOCX)
