# Supplementary material for: Impacts of Population Structure and Analytical Models in Genome-Wide Association Studies of Complex Traits in Forest Trees: A Case Study in Eucalyptus globulus
Source: PLoS One. 2013 Nov 25;8(11):e81267. doi: 10.1371/journal.pone.0081267 (PMC3839935; doi:10.1371/journal.pone.0081267)
Supplement: Figure S1 — Distribution of expected heterozigosity ( He ) values for the 2,643 DArT markers genotyped in the E. globulus mapping population (average He = 0.33). (DOCX) [file pone.0081267.s001.docx]

**Figure S1.** Distribution of expected heterozigosity (*H_e_*) values for the 2,643 DArT markers genotyped in the *E. globulus* mapping population (average *H_e_* = 0.33).
