# Supplementary material for: Impacts of Population Structure and Analytical Models in Genome-Wide Association Studies of Complex Traits in Forest Trees: A Case Study in Eucalyptus globulus
Source: PLoS One. 2013 Nov 25;8(11):e81267. doi: 10.1371/journal.pone.0081267 (PMC3839935; doi:10.1371/journal.pone.0081267)
Supplement: Figure S2 — Linkage Disequilibrium (LD) decay as measured by r2 for pairs of DArT markers across the genome (i.e., intra- and inter-chromosomal), against genetic distance (in cM) in the 303 E. globulus trees. The second-degree Loess fitting curve illustrates the LD decay based on the nonlinear regression of r2 on genetic distance while the horizontal line indicate the baseline r2 values based on the 95th percentile of the r2 values distribution. (DOCX) [file pone.0081267.s002.docx]

**Figure S2**. Linkage Disequilibrium (LD) decay as measured by r^2^ for pairs of DArT markers across the genome (i.e., intra- and inter-chromosomal), against genetic distance (in cM) in the 303 *E. globulus* trees. The second-degree Loess fitting curve illustrates the LD decay based on the nonlinear regression of r^2^ on genetic distance while the horizontal line indicates the baseline r^2^ values based on the 95^th^ percentile of the r^2^ values distribution.

**
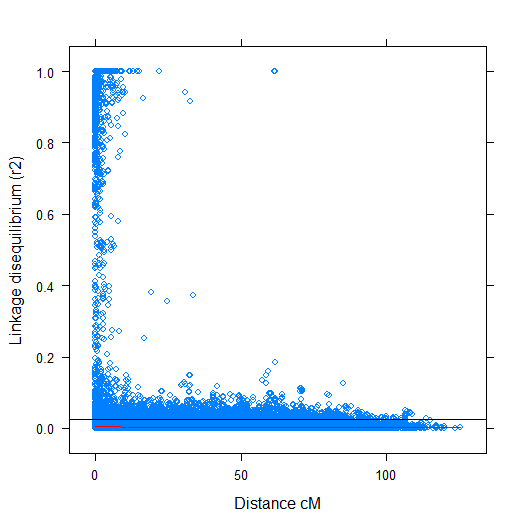
**
