# Supplementary material for: Impacts of Population Structure and Analytical Models in Genome-Wide Association Studies of Complex Traits in Forest Trees: A Case Study in Eucalyptus globulus
Source: PLoS One. 2013 Nov 25;8(11):e81267. doi: 10.1371/journal.pone.0081267 (PMC3839935; doi:10.1371/journal.pone.0081267)

**Figure S3.** Boxplots of growth and wood properties variation in the studied subpopulations. See text for traits’ abbreviation.


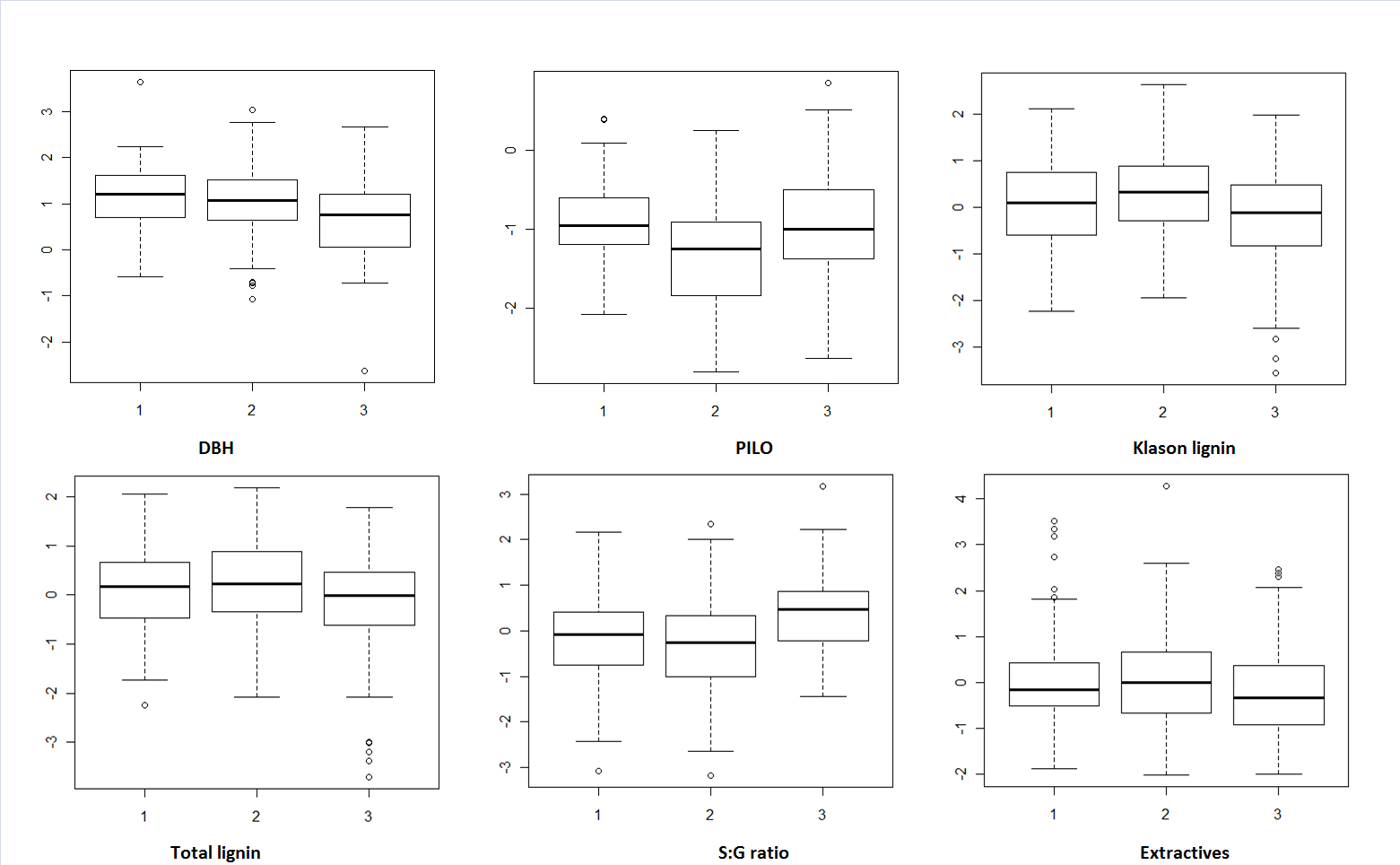

Supplement: Figure S3 — Boxplots of growth and wood properties variation in the studied sub-populations. See text for traits' abbreviation. (DOCX) [file pone.0081267.s003.docx]
