# Supplementary material for: Impacts of Population Structure and Analytical Models in Genome-Wide Association Studies of Complex Traits in Forest Trees: A Case Study in Eucalyptus globulus
Source: PLoS One. 2013 Nov 25;8(11):e81267. doi: 10.1371/journal.pone.0081267 (PMC3839935; doi:10.1371/journal.pone.0081267)
Supplement: Figure S4 — Distribution of pair-wise relatedness coefficients among the 303 E. globulus trees. Values greater than 0.5 are not shown and account for only 0.08% of the distribution. (DOCX) [file pone.0081267.s004.docx]

**Figure S4.** Distribution of pair-wise relatedness coefficients among the 303 *E. globulus* trees. Values greater than 0.5 are not shown and account for only 0.08 % of the distribution.
