# Supplementary material for: Impacts of Population Structure and Analytical Models in Genome-Wide Association Studies of Complex Traits in Forest Trees: A Case Study in Eucalyptus globulus
Source: PLoS One. 2013 Nov 25;8(11):e81267. doi: 10.1371/journal.pone.0081267 (PMC3839935; doi:10.1371/journal.pone.0081267)
Supplement: Table S1 — Significances for the differences of the least square means based on subpopulations estimated by the STRUCTURE software. See text for traits' abbreviation. (DOCX) [file pone.0081267.s005.docx]

**Table S1.** Significances for the differences of the least square means based on subpopulations estimated by the STRUCTURE software. See text for traits’ abbreviation.

| **STRUCTURE**  **Population** | **Trait** | | | | | | |
| --- | --- | --- | --- | --- | --- | --- | --- |
|  | **DBH** | **PILO** | **S:G ratio** | **Klason lignin** | | **Total lignin** | **Extractives** |
| **1** | a | a | b | | a | ab | a |
| **2** | a | b | b | | a | a | a |
| **3** | b | a | a | | b | b | a |
